# The NS4B A119T mutation of dengue virus 2 confers adaptation to mammalian cells and increased pathogenicity in AG129 mice

**DOI:** 10.1099/jgv.0.002358

**Published:** 2026-11-05

**Authors:** Koshiro Tabata, Yukari Itakura, Kentaro Uemura, Malala Mulavu, Miho Yamashita, Takako Matsushita, Shinji Kusakabe, Shinsuke Toba, Michihito Sasaki, Shinji Saito, Gabriel Gonzalez, William W. Hall, Hirofumi Sawa, Akihiko Sato, Yasuko Orba

**Affiliations:** 1 Institute for Vaccine Research and Development (HU-IVReD), Hokkaido University, Sapporo, Japan; 2 Laboratory of Virus Control, Center for Infectious Disease Education and Research (CiDER), The University of Osaka, Osaka, 565-0871, Japan; 3 Drug Discovery and Disease Research Laboratory, Shionogi & Co., Ltd., Osaka, Japan; 4 Division of Molecular Pathobiology, International Institute for Zoonosis Control, Hokkaido University, Sapporo, Hokkaido, 001-0020, Japan; 5 International Collaboration Unit, International Institute for Zoonosis Control, Hokkaido University, Sapporo, Hokkaido, 001-0020, Japan; 6 Global Virus Network, Tampa, Florida, USA; 7 National Virus Reference Laboratory, University College Dublin, Belfield, Dublin, 4, Ireland; 8 One Health Research Center, Hokkaido University, Sapporo, Hokkaido, 001-0020, Japan; 9 Division of Anti-Virus Drug Research, International Institute for Zoonosis Control, Hokkaido University, Sapporo, Japan

**Keywords:** dengue virus, mouse model, AG129, NS4B

## Abstract

Mouse-adapted dengue virus type 2 (DENV2), designated the AG-P10 strain, was established through serial blind passages between AG129 mice and mosquito C6/36 cells. Compared with the parental strain INDIA/09-74, the AG-P10 strain exhibited increased virulence in AG129 mice, leading to rapid body weight loss and earlier mortality. *In vitro* characterization of the AG-P10 strain demonstrated significantly enhanced viral replication and larger focus formation in mammalian Vero and A549 cells but not in mosquito-derived C6/36 cells, suggesting host-specific adaptation. Whole-genome sequencing of the AG-P10 strain identified two nonsynonymous mutations, A119T in NS4B and E802Q in NS5. Through an analysis of recombinant viruses, we determined that the A119T mutation alone is sufficient to confer enhanced pathogenicity in AG129 mice and increased viral replication in Vero and A549 cells, suggesting its potential role in viral adaptation and pathogenesis. The DENV2 with the A119T mutation, as well as the AG-P10 strain, thus provides a robust animal model for developing antiviral therapeutics and vaccines against DENV and for investigating the molecular mechanisms governing DENV2 replication and virulence.

## Data Summary

The complete genome sequences of the recombinant viruses have been deposited in the DNA Data Bank of Japan (DDBJ)/European Nucleotide Archive (ENA)/GenBank databases under accession numbers LC918703, LC918704, LC918705 and LC918706.

## Introduction

Dengue virus (DENV), a member of the mosquito-borne *Orthoflaviviruses* genus in the family *Flaviviridae*, continues to pose a significant global health threat [1]. Approximately 14 million cases and 9,000 deaths were attributed to DENV infections worldwide in 2024 [2]. Most commonly, DENV infection results in an asymptomatic or a mild febrile illness [3, 4]. However, some individuals develop severe manifestations such as dengue haemorrhagic fever and dengue shock syndrome, which are associated with plasma leakage, shock or severe organ dysfunction [5, 6]. Despite the global burden, the development of effective antivirals and vaccines remains challenging, partly due to the lack of robust and representative animal models that closely mimic human symptoms [7].

Most immunocompetent adult mice do not sustain substantial infection or signs of disease by inoculation of clinical DENV isolates because of interferon (IFN)-mediated antiviral responses [8]. Therefore, IFN receptor-deficient mouse strains such as AG129 mice (lacking both IFN-α/β and IFN-γ receptors) have been widely used as DENV infection animal models in dengue research [9–11]. However, clinical DENV isolates often induce neurological symptoms in these models, which are atypical of human disease [12, 13]. To address this, some mouse-adapted DENV strains that cause human-like symptoms in mice without neuroinvasion were established by serially passaging a non-mouse-adapted DENV type 2 (DENV2) strain between mosquito cells and mice [14, 15]. These mouse models infected with mouse-adapted DENV2 have been utilized to develop anti-DENV drugs and vaccines [16–18] and to identify elevated levels of TNF-α as a key contributor to DENV-induced severe disease in mice [19]. However, the specific viral genetic changes responsible for mouse adaptation and increased virulence have not been well characterized.

Following alternate passages between mosquito cells and AG129 mice, we previously established a mouse-adapted DENV2 strain (AG-P10) that exhibited high virulence in AG129 mice without inducing neurological symptoms and used this model for antiviral evaluation [20]. In this study, we conducted a molecular and biological characterization of the AG-P10 strain to elucidate the mechanisms underlying its increased virulence and adaptation to mice. Whole-genome sequencing identified two nonsynonymous mutations: A119T in the non-structural protein (NS) 4B and E802Q in NS5. Using reverse genetics systems and phenotypic analysis of recombinant viruses, we demonstrated that the A119T mutation in NS4B is a key determinant of enhanced viral replication and pathogenicity in mammalian hosts. These findings contribute to a further understanding of DENV host adaptation and provide a valuable model for antiviral and vaccine research as well as for investigating the molecular functions of NS4B in DENV replication.

## Methods

### Cells

*Aedes albopictus*-derived C6/36 mosquito cells (ATCC, Manassas, VA, USA) were maintained in Eagle’s minimum essential medium (MEM, Sigma-Aldrich, Merck, St. Louis, MO, USA) supplemented with 10% FBS and non-essential amino acids at 28 ℃ under 5% CO_2_. Vero cells (Vero9013; JCRB Cell Bank, Osaka, Japan) and A549 cells (RIKEN BRC, Tsukuba, Japan) were maintained in Dulbecco’s modified Eagle’s medium (DMEM, Sigma-Aldrich) supplemented with 10% FBS at 37 °C and 5% CO_2_.

### Animal experiments

Eleven to 12-week-old female AG129 mice, which lack both IFN-α/β and IFN-γ receptors, were inoculated with 100 µl of PBS with or without 1.0×10^5^ f.f.u. of DENV2 via intraperitoneal (ip) route. All mice were monitored daily for 21 days for body weight changes. The humane endpoint was defined as a 25% decrease in body weight.

### Establishment of mouse-adapted dengue virus type 2

The procedure for mouse adaptation of DENV2 hu/INDIA/09-74 strain (INDIA/09-74, GenBank accession no. LC367234) was described previously [20]. In brief, DENV2 INDIA/09-74 strain was propagated in C6/36 cells and titrated by plaque-forming assay. Then, 7-week-old IFN-α/β and -γ receptor-deficient female AG129 mice (purchased from Marshall BioResources, East Yorkshire, UK, and bred in-house) were inoculated ip with 4.0×10^5^ p.f.u. of DENV2. After 3 days post-infection (dpi), mice were euthanized under deep anaesthesia by isoflurane inhalation, followed by serum collection. Following propagation of the viruses in C6/36 cells for 7 days, the cell supernatant was passaged to AG129 mice in the same manner. This adaptation process was repeated 10 times, and the passaged virus was used in subsequent experiments as the AG-P10 strain.

### Next-generation sequencing analysis

Viral RNA was extracted using the QIAamp Viral RNA Mini kit (Qiagen, Hilden, Germany) according to the manufacturer’s instructions, and cDNA was synthesized using PrimeScript II High Fidelity One Step reverse transcription polymerase chain reaction (RT-PCR) (Takara Bio, Shiga, Japan). DNA libraries were prepared using the Nextera XT DNA Library Preparation Kits (Illumina, San Diego, CA, USA). Next-generation sequencing (NGS) was conducted on an iSeq 100 System (Illumina), and the sequences were analysed using CLC Genomics Workbench ver. 21.0.3 software (Qiagen).

### Focus-forming assays

Vero cells (1.5×10^5^ cells/well), A549 cells (1.8×10^5^ cells/well) or C6/36 cells (6.0×10^5^ cells/well) in 24-well plates were inoculated with each virus and incubated for 1 h at 37 °C (Vero cells and A549 cells) or 28 °C (C6/36 cells). For virus titration, C6/36 were used and inoculated with 10-fold serial dilutions of the viruses. After incubation, the inocula were removed and overlay media (MEM containing 0.5% methyl cellulose and 2% FBS) was added to the cells. At 72 hours post-infection (hpi), the cells were fixed with prechilled methanol at −30 °C for 10 min. The fixed cells were then stained with the anti-flavivirus NS1 recombinant mouse IgG antibody clone 1G5.3 [21] in PBS containing 1% BSA at room temperature (RT) for 1 h. After three washes with PBS, Alexa Fluor 488-conjugated anti-mouse IgG antibody (Invitrogen, Waltham, MA, USA) was applied as a secondary antibody at RT for 1 h. Viral focus images were acquired using a fluorescence microscope (IX73, Olympus, Tokyo, Japan). Focus size was measured using an imaging software cellSens (Olympus). For virus titration, virus titres were calculated based on the number of foci and expressed as f.f.u. ml^−1^.

### Growth kinetics

Vero cells (2.0×10^5^ cells/well), A549 cells (4.0×10^5^ cells/well) or C6/36 cells (3.0×10^5^ cells/well) in six-well plates were inoculated with each virus at an m.o.i. of 0.1 for Vero cells and C6/36 cells and at an m.o.i. of 1 for A549 cells and incubated for 1 h at 37 °C (Vero cells and A549 cells) or 28 °C (C6/36 cells). After incubation, the cells were washed twice with PBS, and 3 ml of fresh MEM containing 2% FBS was added. Supernatants (200 µl) were harvested at the time points indicated in each figure, and virus titres in each supernatant were measured by a focus-forming assay (FFA).

### Reverse genetics of recombinant DENV2

To generate the recombinant DENV2, we used the circular polymerase extension reaction (CPER) method based on a previous report [22]. In short, viral genome RNA was extracted from supernatant of cell cultures by TRIzol LS (Invitrogen) and Direct-zol RNA MiniPrep kits (Zymo Research, Irvine, CA, USA). To generate template DNA for CPER PCR, five cDNA fragments encoding the DENV2 genome were amplified with PrimeSTAR GXL DNA polymerase (Takara Bio) and cloned into the pMW119 DNA vector (NIPPON GENE, Tokyo, Japan). Point mutations A119T (G to A at nt position 7084) in NS4B and E802Q (G to C at nt position 9877) in NS5 were introduced by PCR-based mutagenesis. A linker fragment encoding a polyadenylation signal, hepatitis delta virus ribozyme and OpiE2 promoter was also synthesized. CPER fragments were then amplified from the plasmids encoding the viral genome cDNA fragments and the linker fragment by PCR using primers containing overlap regions of at least 20 bases with the adjacent fragments. These DNA fragments were subsequently assembled into a circular DNA by CPER and directly transfected into C6/36 cells by TransIT-Insect Transfection Reagent (Mirus bio, Merck, Madison, WI, USA). One week after transfection, the supernatants of the cells were harvested as P0 stock and passaged onto C6/36 cells for virus propagation. At 7 dpi, the supernatants were harvested and stored as working stocks. Virus titres from the working stocks were measured by FFA. All genomes of recombinant viruses were validated by NGS.

### Attachment and entry assays

The attachment and entry assays were conducted following procedures described previously [23]. Briefly, Vero cells were treated with virus at m.o.i.=10 for 2 h at 4 and 37 °C for the attachment and entry assays, respectively, followed by three washes with PBS. Subsequently, RNA was extracted from the cells immediately after the incubation for the attachment assay. For the entry assay, cells were then treated with trypsin for 3 min at 37 °C to remove virions on the cell surface and suspended in DMEM containing 10% FBS. The cells were collected by centrifugation and RNA was extracted from the cells. RNA was subjected to quantitative reverse transcription-PCR (qRT-PCR) to determine viral genome copy numbers.

### qRT-PCR

qRT-PCR was performed using One Step TB Green PrimeScript PLUS RT-PCR Kit (Perfect Real Time) (Takara Bio) and primer sets targeting DENV NS5 (F: 5ʹ-AGT GGA CAC GAG AAC CCA AGA-3ʹ, R: 5ʹ-TTC GGC CGT GAT TTT CAT TAG-3ʹ), β-actin (F: 5ʹ-CAT GTA CGT TGC TAT CCA GGC-3ʹ, R: 5ʹ-CTC CTT AAT GTC ACG CAC GAT-3ʹ), human IFN-β (F: 5ʹ-ATT GCT CTC CTG TTG TGC TT-3ʹ, R: 5ʹ-TCT CCT CAG GGA TGT CAA AGT-3ʹ), human IFIT3 (F: 5ʹ-CAG AGG GCA GTC ATG AGT GAG G-3ʹ, R: 5ʹ-ATC TTC TAG ATC CCT TGA GAC ACT G-3ʹ), human OAS1 (F: 5ʹ-AGT TGA CTG GCG GCT ATA AAC-3ʹ, R: 5ʹ-GTG CTT GAC TAG GCG GAT GAG-3ʹ) and human GAPDH (F: 5ʹ-CTG ACT TCA ACA GCG ACA CC-3ʹ, R: 5ʹ-TAG CCA AAT TCG TTG TCA TAC C-3ʹ) following the manufacturer’s procedure [24].

### Statistical analysis

All statistical analyses were performed using GraphPad Prism software version 8.0. The methods of statistical analysis are described in the figure legends for each experiment.

## Results

### Fitness of AG129 mouse-adapted DENV2 increased in mammalian cells

AG129 mouse-adapted DENV2 INDIA/09-74 (AG-P10) strain was obtained through 10 blind passages in AG129 mice and C6/36 cells (Fig. 1a). To evaluate the pathogenicity of the AG-P10 strain, AG129 mice were inoculated ip with 1.0×10^5^ f.f.u. of the virus and monitored daily for body weights and survivals. Infection with the parental INDIA/09-74 strain exhibited bimodal changes in body weight in all AG129 mice (Figs 1b and S1, available in the online Supplementary Material). During the second reduction in body weight, hind limb paralysis was observed. On the other hand, body weights of mice infected with AG-P10 strain rapidly dropped to the humane endpoint without neurological symptoms (Figs 1b and S1). All mice infected with AG-P10 strain died within 7–8 dpi, whereas mice infected with the parental INDIA/09-74 strain had a survival period ranging from 9 to 18 days (Fig. 1c). To evaluate the *in vitro* characteristics of the AG-P10 strain, the viral growth kinetics in mammalian-derived Vero cells, A549 cells and mosquito-derived C6/36 cells were compared with those of the parental strain. Notably, the AG-P10 strain exhibited significantly higher replication in both Vero and A549 cells at 1 and 2 dpi compared to the parental virus, while there were no differences in viral growth in C6/36 cells at any time points (Fig. 1d). Moreover, the AG-P10 strain formed significantly larger foci in Vero and A549 cells than the parental strain at 3 dpi (Fig. 1e–h). These results suggest that the AG-P10 strain had acquired higher virulence in AG129 mice due to increased replication in mammalian cells.

**Fig. 1.**
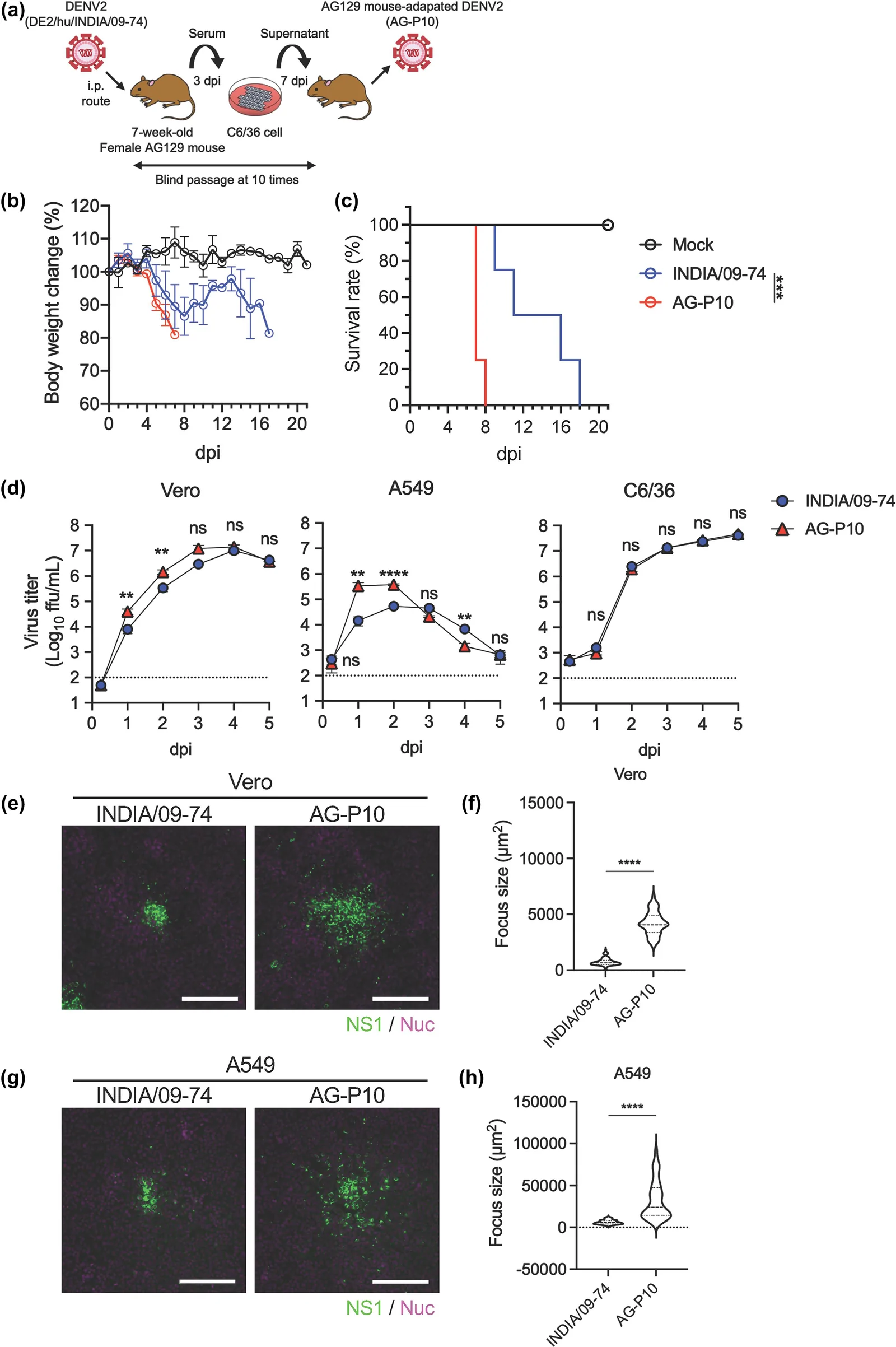
AG129-adapted DENV2 (AG-P10) was developed and characterized *in vivo* and *in vitro*. (**a**) Schematic illustration of AG129 adaptation of DENV2. (**b and c**) AG129 mice were inoculated with either mock control (*n*=3), the parental DENV2 INDIA/09-74 strain (*n*=4) or the mouse-adapted AG-P10 (*n*=4) and then monitored daily for changes in body weight (BW) (**b**) and survival rate (**c**). The BW changes are expressed as the mean±sd of biological replicates. Statistical analysis was performed using the log-rank test between the parental DENV2 and the AG-P10 (***: *P*<0.001). (**d**) Growth kinetics in mammalian Vero cells (m.o.i.=0.1) and A549 cells (m.o.i.=1) and mosquito-derived C6/36 cells (m.o.i.=0.1) were compared between the parental DENV2 and AG-P10. Values are expressed as the mean±sd of biological replicates (*n*=3). The dotted line indicates the limit of detection (LOD). Statistical analysis was performed by multiple t-test. (**: *P*<0.01). (e–h) Cell-to-cell infectivity was compared between the parental DENV2 and AG-P10. (**e and g**) Representative focus images of each DENV2 in (**e**) Vero cells and (**g**) A549 cells are presented. Viral antigen NS1 and nuclei are shown in green and magenta, respectively. Scale bars indicate 500 µm. (**f and h**) Focus sizes were measured in randomly selected foci in (**f**) Vero cells and (**h**) A549 cells, and the mean focus size was compared between the parental DENV2 (Vero: *n*=95; A549: *n*=49) and AG-P10 (Vero: *n*=47; A549: *n*=37). Statistical analysis was performed by the Mann–Whitney U test (****: *P*<0.0001).

### A119T mutation in the NS4B was responsible for AG129 mouse adaptation of the AG-P10 strain

To determine the responsible mutations for the mammalian adaptation of the AG-P10 strain, its whole-genome sequence was analysed by NGS. Two mutations, A119T and E802Q, were identified in NS4B and NS5, respectively (Fig. 2a). For further functional analysis of the mutations, we generated recombinant viruses using the CPER method: the DENV2 INDIA/09-74 strain (rWT) and mutant clones with a single mutation (either A119T or E802Q) or a double mutation (A119T+E802Q). We confirmed that the biological characteristics of DENV2-rWT and DENV2-A119T+E802Q in Vero cells were similar to those of each parental virus; INDIA/09-74 and AG-P10 strain, respectively (Fig. S2A and B).

**Fig. 2.**
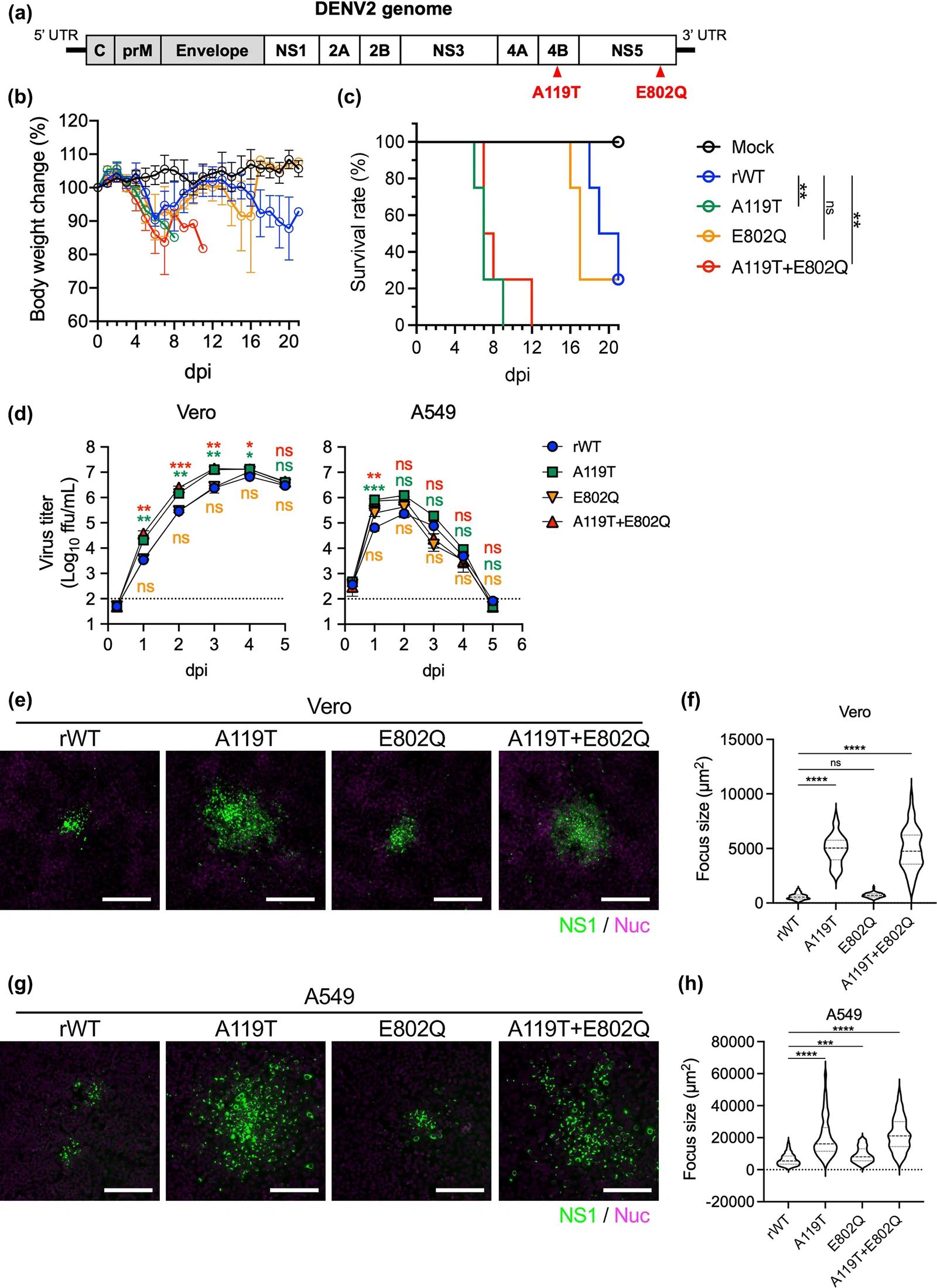
The A119T mutation in NS4B enhances DENV2 lethality in mice and improves its *in vitro* replication properties. (**a**) Schematic illustration of the viral genome of DENV2 and amino acid substitutions from AG-P10. (**b and c**) AG129 mice were inoculated with either mock control (*n*=3), recombinant DENV2 wild-type (rWT) (*n*=4), mutants with A119T (*n*=4), E802Q (*n*=4) or A119T+E802Q (*n*=4) and then monitored daily for changes in BW (**b**) and survival rate (**c**). The BW changes are expressed as the mean±sd of biological replicates. Statistical analysis was performed using the log-rank test compared to the recombinant DENV2-rWT. (**: *P*<0.01) (**d**) Growth kinetics of the recombinant DENV2 mutants (m.o.i.=1) in Vero cells and A549 cells were compared. Values are expressed as the mean±sd of biological replicates (*n*=3). The dotted line indicates the LOD. Statistical analysis was performed by multiple t-test, comparing each group to DENV2-rWT. (*: *P*<0.05, **: *P*<0.01, ***: *P*<0.001, ns: not significant.) (e–h) Focus formation was compared among recombinant DENV2. (**e and g**) Representative focus images of each DENV2 in (**e**) Vero cells and (**g**) A549 cells are presented. Viral antigen NS1 and cell nuclei are shown in green and magenta, respectively. Scale bars indicate 500 µm. (**f**) Focus sizes were measured from randomly selected foci in (**f**) Vero cells and (**h**) A549 cells, and the mean focus size was compared between the DENV2-rWT (Vero: *n*=63; A549: *n*=127), mutant with A119T (Vero: *n*=47; A549: *n*=105), E802Q (Vero: *n*=76; A549: *n*=139) and A119T+E802Q (Vero: *n*=74; A549: *n*=119). Statistical analysis was performed by the Mann–Whitney U test. (****: *P*<0.0001).

To evaluate the pathogenicity of the recombinant viruses, AG129 mice were inoculated ip with these viruses and monitored daily for body weight changes and survivals. Similar to the parental INDIA/09-74 strain, both DENV2-rWT and DENV2-E802Q infections showed bimodal body weight changes in mice (Figs 2b and S2C), resulting in 80% mortality within 3 weeks (Fig. 2c). On the other hand, both DENV2-A119T and DENV2-A119T+E802Q infections of AG129 mice resulted in similar body weight changes and survival rate to those observed for the AG-P10 strain. Importantly, the survival curves differed significantly between mice infected with DENV2 recombinants with and without the A119T mutation (Fig. 2c). Furthermore, we compared the viral growth kinetics and focus sizes in Vero and A549 cells among these DENV2 recombinants. Both DENV2 recombinants with the A119T mutation exhibited significantly higher virus titres at 1–4 dpi in Vero cells and at 1 dpi in A549 cells than DENV2-rWT (Fig. 2d). In addition, the DENV2 recombinants carrying the A119T mutation showed significantly larger focus sizes than those without A119T (Fig. 2e–h) in both cell lines. DENV2-E802Q exhibited no significant difference in focus size in Vero cells, whereas a significant increase was observed in A549 cells compared with the DENV2-rWT (Fig. 2e-h). These results suggest that the A119T mutation in NS4B is essential for the adaptation of the AG-P10 strain to AG129 mice and contributes to increased viral fitness in mammalian cells.

### The A119T mutation in NS4B promoted the early phase of DENV2 replication cycle without suppressing host innate immune responses in mammalian cells

To elucidate the viral replication steps in mammalian cells that are affected by the mutations A119T and E802Q acquired by the AG-P10 strain, replication dynamics of the recombinant virus clones at early phases were assessed in Vero and A549 cells. Since mutations in NS4B, a NS that forms the replication complex, may affect viral genome replication, we investigated the dynamics of the recombinant viruses during the early phase of virus replication cycle in Vero cells.

Both DENV2-A119T and DENV2-A119T+E802Q showed significantly higher viral genome replication in Vero and A549 cells at 24 hpi than DENV2-rWT and DENV2-E802Q (Fig. 3a). In contrast, no significant differences were observed among the four recombinant clones during virus attachment and entry in Vero cells (Fig. S3). These results suggest that the A119T mutation in NS4B enhances viral genome replication during the early phase of the virus replication cycle in mammalian cells.

**Fig. 3.**
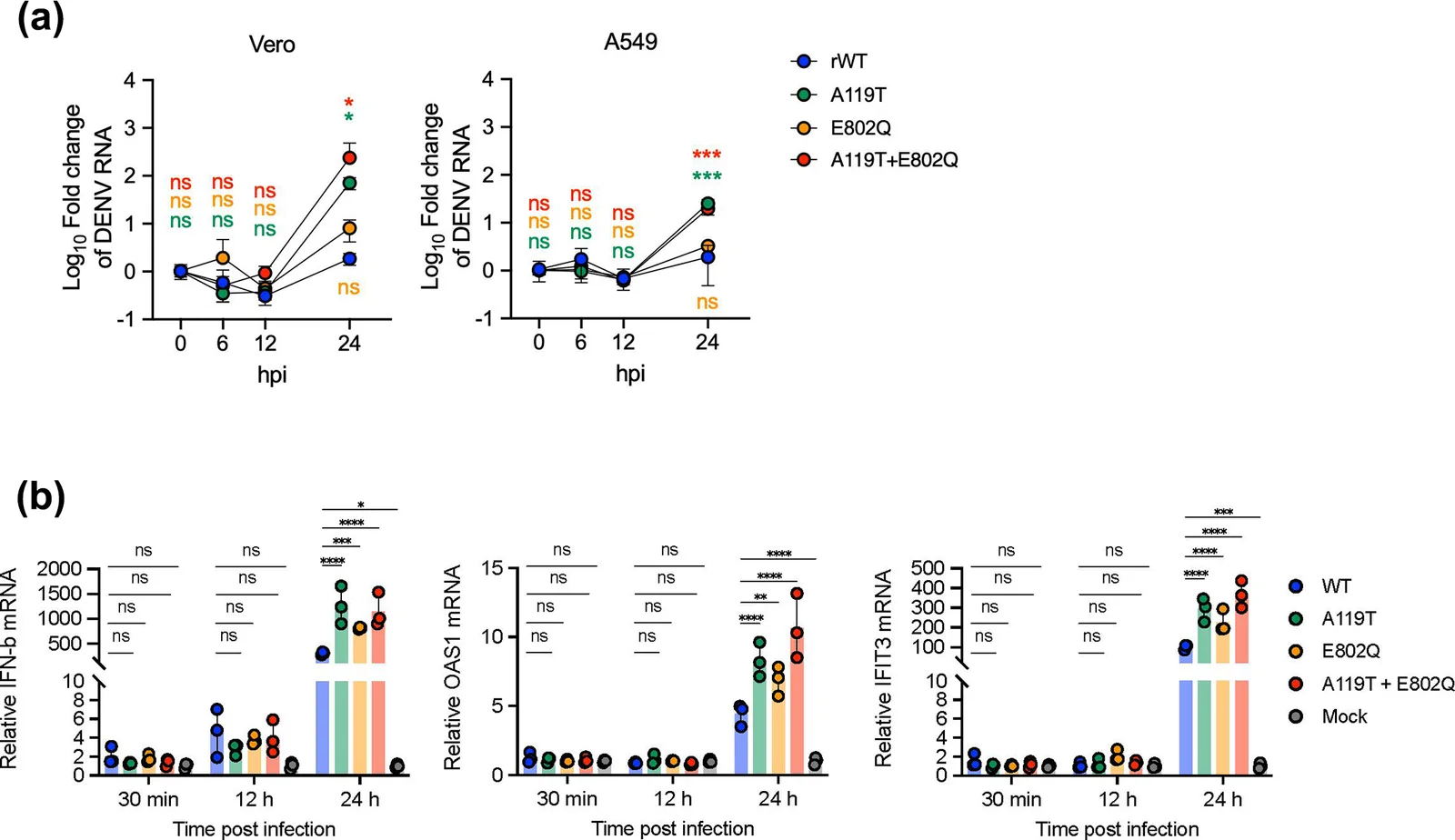
The A119T mutation enhances virus replication during the early phase of the DENV2 replication cycle without suppressing host innate immune responses. (**a**) Viral replication during the early phase. Viral genome replication in Vero cells (m.o.i.=0.1) and A549 cells (m.o.i.=1) was quantitated by qRT-PCR. RNA was extracted from cells infected with recombinant DENV2, and viral RNA was quantified by qRT-PCR using the ΔΔCt method, with *β-actin* and *GAPDH* as the reference genes for Vero cells and A549 cells, respectively. Values are normalized to those of 0 hpi and expressed as the mean±sd of biological replicates (*n*=3). Statistical analysis was performed by multiple t-test, comparing each group with DENV2-rWT. (*: *P*<0.05, ***: *P*<0.001, ns: not significant.) (**b**) Host innate immune responses were assessed in A549 cells. RNA was extracted from cells infected with recombinant DENV2 (m.o.i.=1), and the mRNA levels of *IFN-β*, *OAS1* and *IFIT3* were quantified by qRT-PCR using the ΔΔCt method, with *GAPDH* as the reference gene and values normalized to those of the mock control. Bars represent means, and dots represent individual values (*n*=3). Statistical analysis was performed using two-way ANOVA with Dunnett’s multiple-comparison test, comparing each group to DENV2-rWT. (*: *P*<0.05, **: *P*<0.01, ***: *P*<0.001, ****: *P*<0.0001).

To further clarify whether this enhanced replication involves suppression of host innate immunity, a known function of NS4B in mammalian cells [25–27], we evaluated expression of IFN and IFN-stimulated genes (ISGs) in IFN-competent A549 cells infected with the recombinant viruses. A previous study reported that NS4B mutations associated with adaptation to human cells suppress innate immune responses as early as 0.5 hpi, whereas this suppression is no longer apparent at 24 hpi [26]. Based on these findings, samples were collected at 0.5, 12 and 24 hpi to assess the effects of NS4B mutations on innate immune responses. At 24 hpi, mRNA levels of *IFN-β* and downstream ISGs, including *OAS1* and *IFIT3*, were significantly upregulated in cells infected with all three mutant viruses relative to DENV2-rWT, with a tendency towards higher expression in cells infected with viruses carrying the A119T mutation, whereas no significant difference was observed among these viruses at 0.5 and 12 hpi (Fig. 3b). These innate immune responses were associated with viral replication (Fig. 3a). Collectively, these findings indicate that the A119T mutation did not suppress the induction of host innate immune responses in A549 cells. Thus, the enhanced replication associated with the A119T mutation is unlikely to be attributable to the suppression of innate immune responses.

## Discussion

In a previous study, we established a mouse-adapted DENV2 strain, AG-P10, through serial passages between mosquito-derived cells and IFN-α/β/γ receptor-deficient AG129 mice [20]. To understand the adaptation, characterization of the AG-P10 strain was performed in this study. Whole-genome sequencing of the AG-P10 strain revealed two nonsynonymous mutations, A119T in NS4B and E802Q in NS5, acquired during the adaptation process (Fig. 2a). Functional analysis indicated that the A119T mutation in NS4B plays a pivotal role in the adaptation to mice (Fig. 2b and c). Furthermore, this mutation enhanced viral replication, particularly during the early phase of the virus replication cycle in mammalian Vero cells and A549 cells (Figs 2d-h and 3a). Previously, Xie *et al*. demonstrated that the A119T mutation in NS4B, in combination with P104L, conferred resistance to the anti-NS4B compound NITD-618 by promoting viral replication in human A549 cells but not in mosquito-derived C6/36 cells [28]. Consistently, another study reported that a single amino acid substitution (L52F) in DENV2 NS4B enhanced viral RNA synthesis and increased virulence in AG129 mice [25]. These observations, together with our findings, emphasize that adaptive mutations in NS4B can modulate viral replication efficiency and thereby contribute to mammalian adaptation and pathogenicity. Overall, our results suggest that the A119T mutation in NS4B either directly or cooperatively enhances efficiency during the early phase of virus replication in mammalian cells. Consequently, DENV carrying the A119T mutation may replicate more rapidly and exhibit pathogenicity earlier in immunodeficient mice than the parental strain.

Additionally, our group has previously reported that DENV2 with a G124A mutation in NS4B was obtained by serial passage in baby hamster kidney-21 cells [29]. The rDENV2-G124A exhibited enhanced growth in mammalian-derived Vero and Huh-7 cells. In contrast, the rDENV2-G124A lost its focus-forming ability in mosquito-derived C6/36 cells, although viral genomic RNA remained detectable. Interestingly, both A119T and G124A mutations are located within the third transmembrane domain (TMD3) of NS4B. The TMD3 region of NS4B is implicated in the inhibition of host IFN signalling [26, 27]. A previous study reported that DENV1 clinical isolates acquired V116A or V116M mutations in the TMD3 of NS4B during adaptation to human cells [25]. The V116A or V116M mutations enhanced the inhibitory function of TMD3 against IFN signalling and increased viral replication in mammalian cells except for IFN-deficient Vero cells. Our results demonstrated that the A119T mutation increased virus propagation even in the absence of IFN responses (Figs 2b–f and 3a), as both Vero cells and AG129 mice lack functional IFN signalling [30, 31]. Furthermore, viruses carrying the A119T mutation did not suppress innate immune responses in IFN-competent A549 cells (Fig. 3b). Collectively, these findings indicate that the A119T mutation enhances viral virulence and propagation in mammalian hosts by intrinsically optimizing replication efficiency rather than through immune evasion. However, the exact underlying molecular events responsible for this enhancement remain unclear. Previous studies have suggested that NS4B contributes to viral replication either by directly facilitating replication complex assembly or by endoplasmic reticulum membrane remodelling to form viral replication factories that protect viral RNA intermediates from host innate immune sensing [32]. Thus, the precise molecular mechanism by which the A119T mutation enhances viral replication remains to be determined. Strand-specific analysis of viral RNA and visualization of replication complex formation will be important for further defining the effect of the A119T mutation on the viral replication process. Such insights will not only deepen our understanding of DENV replication in mammalian hosts but may also identify novel targets for antiviral development. Moreover, our study emphasises the utility of the mouse-adapted DENV2 with a single amino acid mutation A119T in NS4B for the assessment of therapeutics against DENV as well as vaccine development.

## Supplementary material

10.1099/jgv.0.002358 Supplementary Material 1.

## Abbreviations

CPER: circular polymerase extension reaction
DDBJ: DNA Data Bank of Japan
DENV2: dengue virus type 2
DENV: dengue virus
DMEM: Dulbecco’s modified Eagle's medium
dpi: days post-infection
ENA: European Nucleotide Archive
FFA: focus-forming assay
hpi: hours post-infection
IFN: interferon
ip: intraperitoneal
ISG: interferon-stimulated genes
MEM: minimum essential medium
NGS: next-generation sequencing
NS: non-structural protein
qRT-PCR: quantitative reverse transcription polymerase chain reaction
RT: room temperature
RT-PCR: reverse transcription polymerase chain raction
TMD3: third transmembrane domain
