# Supplementary material for: The NS4B A119T mutation of dengue virus 2 confers adaptation to mammalian cells and increased pathogenicity in AG129 mice
Source: J Gen Virol. 2026 Nov 5;107(11):002358. doi: 10.1099/jgv.0.002358 (PMC13739289; doi:10.1099/jgv.0.002358)
Supplement: Supplementary file 1 [file jgv-107-02358-s001.pdf]

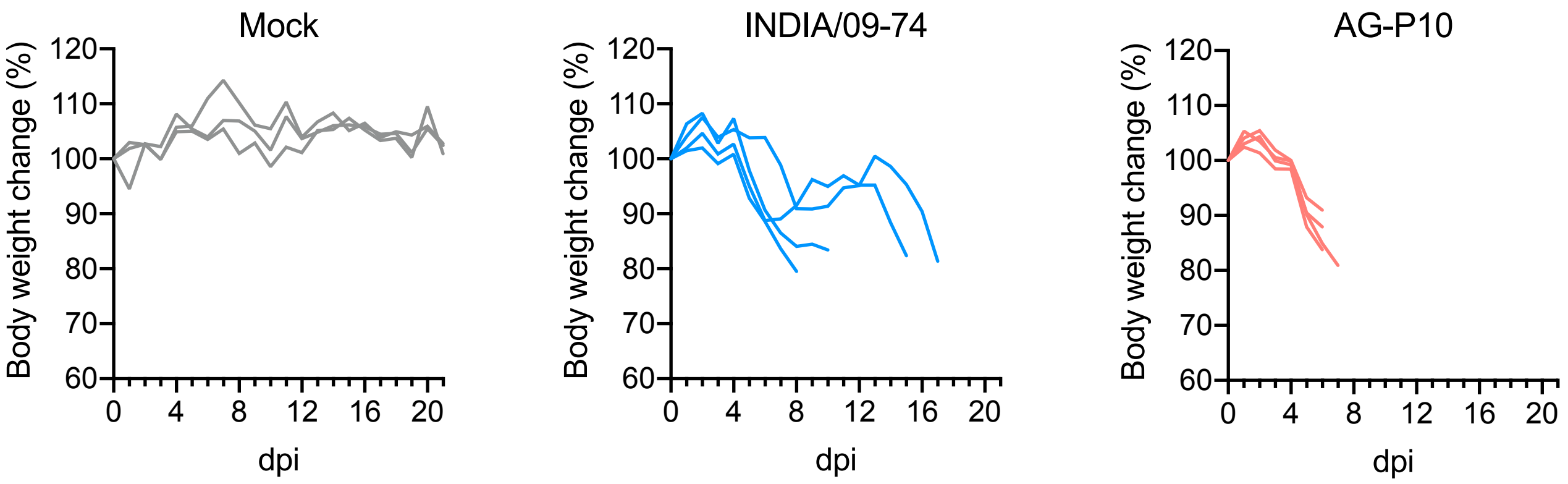

Fig. S1 Individual body weight changes in AG129 mice infected with parental Dengue virus 2 (DENV2) INDIA/09-74 strain, and AG129 mouse-adapted DENV2 AG-P10 strain.
AG129 mice were inoculated with either mock control (n = 3), the parental DENV2 INDIA/09-74 strain (n = 4), or the mouse-adapted AG-P10 (n = 4) and then monitored daily for changes in body weight (BW).

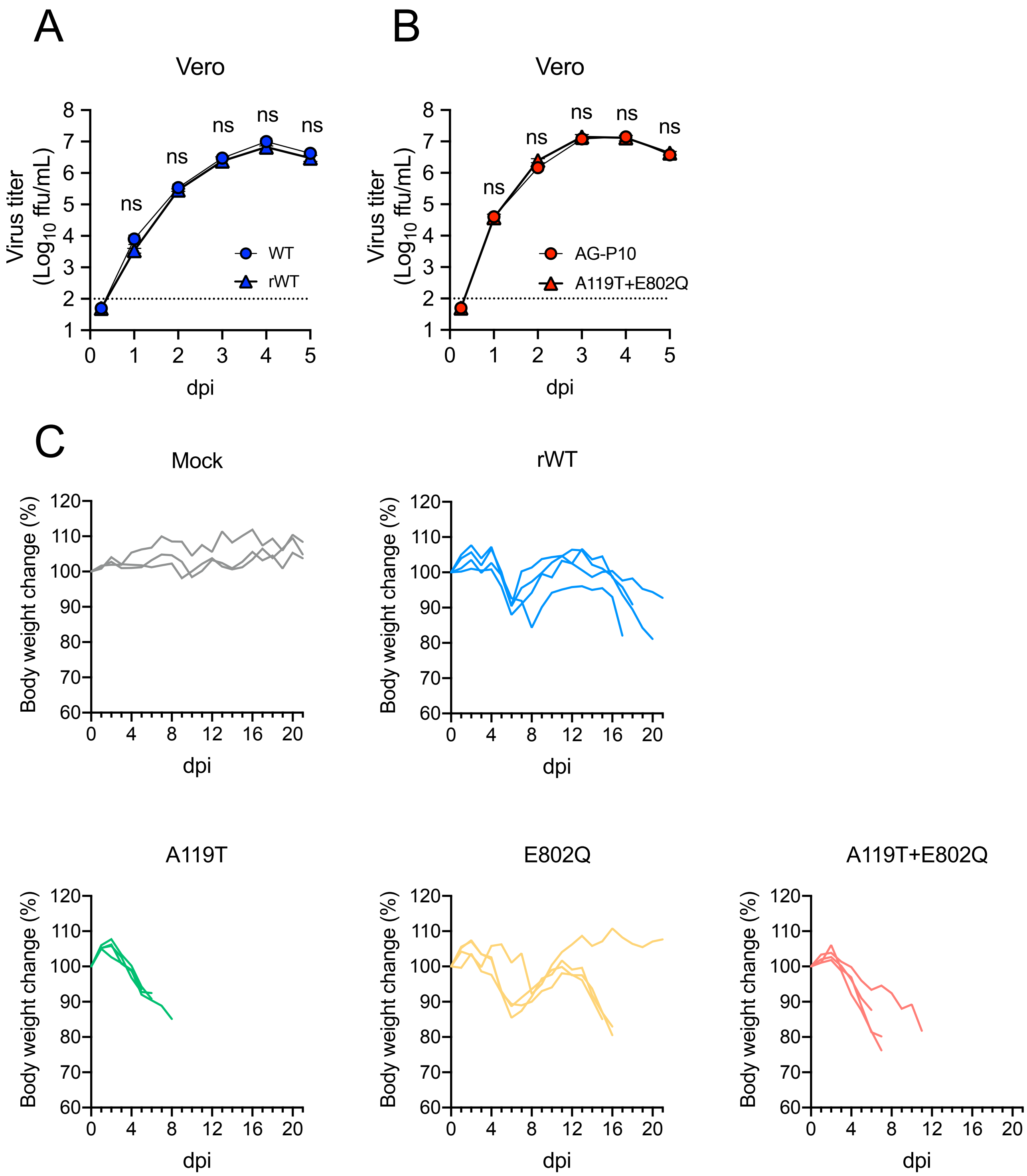

Fig. S2 ***In vitro*** **or** ***in vivo*** **characterization for recombinant DENV2 clones with each mutation. (A and B)** Growth kinetics of (A) parental DENV2 wild type (WT) and recombinant DENV2 WT (rWT) and (B) AG129 mouse-adapted DENV2 (AG-P10) and recombinant DENV2 with A119T/E802Q mutations in Vero cells were compared. Values are expressed as the mean ± SD of biological replicates (n = 3). The dotted lines indicate the limit of detection (LOD). Statistical analysis was performed by the Mann-Whitney U test. **(C)** AG129 mice were inoculated with either mock control (n = 3), DENV2 rWT (n = 4), recombinant DENV2 with A119T (n = 4), with E802Q (n = 4), or with A119T+E802Q and then monitored daily for changes in BW.

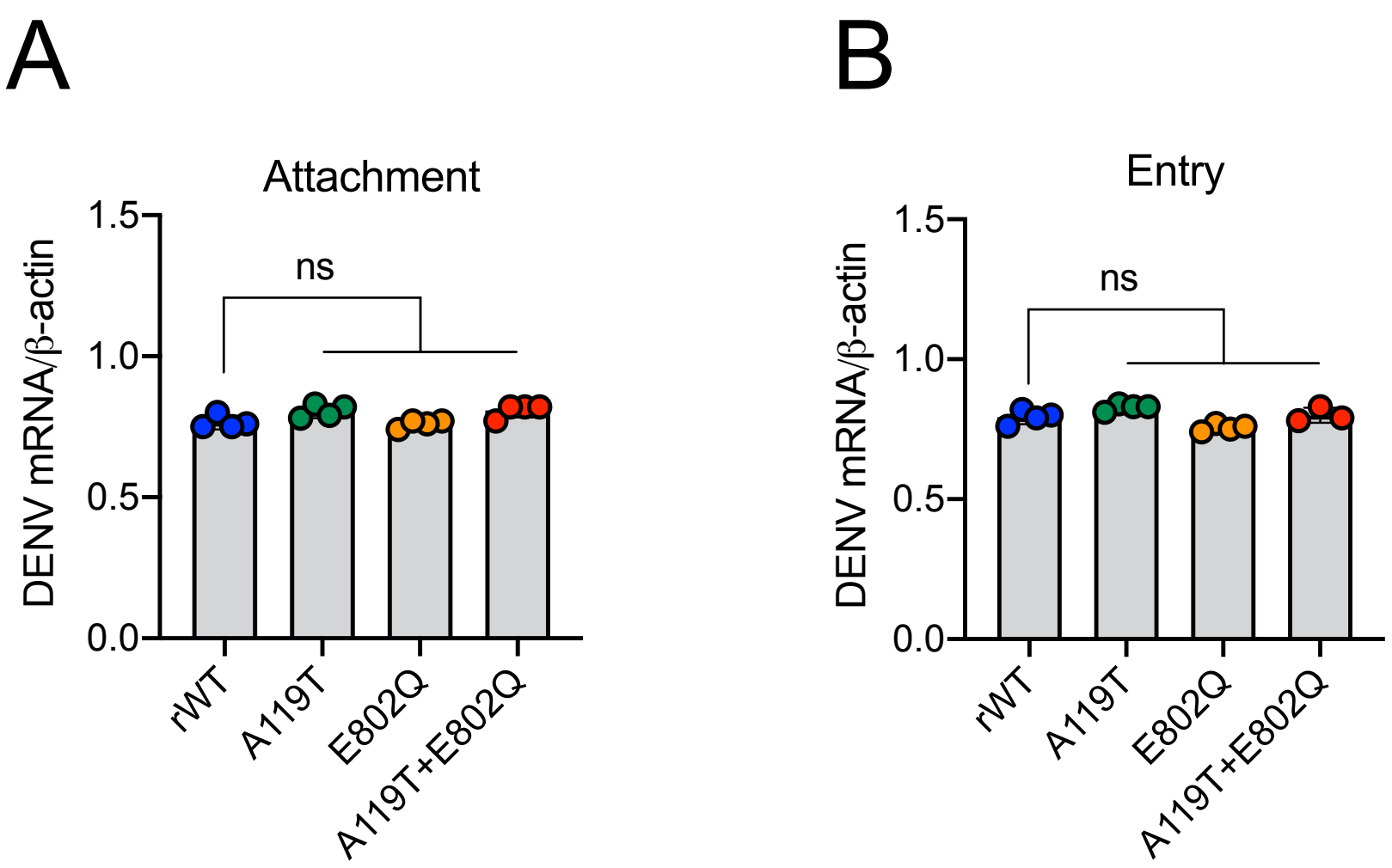

Fig. S3 The A119T mutation does not affect viral attachment or entry.
(A) Virus attachment on the surface of Vero cells was evaluated. The cells were inoculated with recombinant DENV2 and incubated at 4°C for 2 h. After washing the cells, viral RNA was extracted and quantified by qRT-PCR. **(B)** Virus entry was evaluated. Cells were incubated with recombinant DENV2 at 37 °C for 2 h. After incubation, the cells were washed and treated with trypsin, and then viral RNA was extracted and quantitated by qRT-PCR. Statistical analysis was performed by multiple *t*-test, comparing each group to DENV2-rWT. (*: $p < 0.05$, ***: $p < 0.001$)
